# Plant Cadmium Toxicity and Biomarkers Are Differentially Modulated by Degradable and Nondegradable Microplastics in Soil

**DOI:** 10.3390/toxics12070473

**Published:** 2024-06-29

**Authors:** Jun Liu, Zihan Yu, Ningning Song, Haiying Zong, Fangli Wang, Rui Guo, Shaojing Li

**Affiliations:** 1 School of Resources and Environment, Qingdao Agricultural University, Qingdao 266109, China; liujun64@126.com (J.L.); yuzihanhhxx@163.com (Z.Y.); snn05@163.com (N.S.); u571zhy@126.com (H.Z.); flwang0603@163.com (F.W.); 2 Technical Centre for Soil, Agriculture and Rural Ecology and Environment, Ministry of Ecology and Environment, Beijing 100012, China; 3 College of Science and Information, Qingdao Agricultural University, Qingdao 266109, China

**Keywords:** combined pollution, microplastics, biodegradable microplastics, heavy metals, antioxidant system

## Abstract

The impact of microplastics (MPs) as emerging pollutants on plant heavy metal toxicity has been extensively reported in vegetable–soil systems over recent years. However, little attention has been given to cultivar variations between degradable and non-degradable MPs. This study investigated the effects of degradable polylactic acid (PLA) and nondegradable polypropylene (PP) MPs on plant growth and biomarker (malonaldehyde (MDA) and antioxidant enzymes) performance in Cd-contaminated arable soil. The results show that both types of MPs significantly impacted plant biomass and biomarker contents across all three Cd levels. The degree of impact was significantly sensitive to both the type and dose of MPs, as they reduced the soil pH and cation exchange capacity (CEC) while increasing soil dissolved organic carbon (DOC), microbial biomass carbon, and nitrogen. PP exhibited greater root growth inhibition and phytotoxicity at higher doses of 1% and 5% compared to PLA. Specifically, the highest MDA contents were 1.44 and 2.20 mmol mg^−1^ protein for shoots and roots, respectively, in the 5% PLA treatment under a 10.1 mg kg^−1^ Cd level, which were 1.22 and 1.18 times higher than those in corresponding treatments of 5% PP. Overall, PLA had less significant effects on plant phytotoxicity, Cd availability, and soil properties compared to PP. Regression pathway analysis indicated that MPs increased shoot Cd uptake by altering both soil physical–chemical and microbial characteristics. Among the soil variables, pH, CEC, and Cd bioavailability were found to play vital roles. Yet, no single variable acts alone in the mechanism for plant Cd uptake. PLAs are suggested to replace conventional non-biodegradable plastics to control environmental MP pollution, particularly in agricultural systems with higher Cd contamination. However, the long-term effects of the by-products generated during the biodegradation process require further investigation.

## 1. Introduction

Microplastics (MPs are plastics less than 5 mm in size) have raised worldwide public concern due to their ubiquitous presence and potential toxicity to human health [1]. High levels of MPs in agricultural soils have been reported in numerous regions [2,3]. Numerous studies have indicated the negative impact of MPs on the sustainability of agroecosystems and organism health [4,5], thus highlighting the seriousness of their presence. In recent decades, MPs have been increasingly recognized as vectors for coexisting contaminants, thereby increasing their accumulation in both aquatic and terrestrial systems [6]. Previous studies have reported that the increased accumulation of coexisting contaminants in organisms is attributed to the higher toxicity induced by MPs [7]. MPs can also alter crop plant performance, as well as soil physicochemical and microbial properties, thus further impacting the toxicity of coexisting contaminants in soil–plant systems [8]. However, despite the wide presence of MPs in agrosystems, the underlying mechanisms remain largely unexplored.

The impacts of MPs are highly dependent on their polymer type, size/shape, and concentration [9]. MPs, primarily composed of synthetic polymers, are produced from commercial plastics such as polypropylene (PP), polyethylene (PE), and polyvinyl chloride (PVC), all of which are nondegradable [1]. The large specific surface area and colloidal size of nondegradable MPs enable them to sorb coexisting contaminants and potentially serve as transporters, thereby altering the mobility and distribution of these contaminants in soils. Nondegradable MPs have also been proven to negatively affect soil properties such as soil structure, nutrient cycling, and microbial function [8]. To address the soil pollution caused by nondegradable MPs, biodegradable materials such as polylactic acid (PLA) and polypropylene carbonate (PPC) have been introduced, particularly in agricultural activities [10]. Despite the growing use of biodegradable MPs, it remains uncertain whether they are an effective tool for addressing the plastic crisis [11]. For instance, several studies have shown that biodegradable MPs can also act as carriers of various pollutants and exert a toxicity similar to conventionally nondegradable MPs on marine organisms [1,12]. However, there is limited information regarding the influence of biodegradable MPs on soil–plant systems, particularly concerning the underlying mechanisms. Thus, there is an urgent need to explore and contrast the effects of biodegradable and nondegradable MPs on the health of soil–plant systems.

Among the coexisting contaminants with the propensity to interact with both nondegradable and biodegradable MPs, heavy metals stand out as particularly toxic core pollutants due to their inorganic nature [8]. Heavy metal contamination in soils presents a significant global challenge. In 2015, more than 13% of the world’s total cultivated lands, roughly 0.24 billion hectares, were affected by heavy metal contamination [13]. Although MPs have been recognized as emerging pollutants in recent decades, plastics have been in use for nearly 70 years. Consequently, MPs and heavy metals have likely coexisted widely in the environment for a long time. Today, the contamination of MPs and their interactions with heavy metals have garnered global concern [9,14]. The interaction between plastics and heavy metals was reported early in 2010 [15]. Subsequently, numerous studies have indicated the impacts of various types of MPs on the mobility, toxicity, and fractionation of contaminants in different environments [16]. Under stress from pollutants like cadmium (Cd) and MPs, plants generate excessive free radicals and reactive oxygen species, such as malondialdehyde (MDA), thus leading to increased lipid peroxidation of cell membranes [8,17]. Simultaneously, the antioxidant enzyme system, including superoxide dismutase (SOD), peroxidase (POD), and catalase (CAT), is activated to mitigate oxidative stress by scavenging free radicals and detoxifying harmful substances, thereby enhancing plant tolerance and survival capabilities against pollutants [1,9,18]. However, the mechanisms of impact and the combined effects of these interactions are not well understood, particularly in soil–plant systems. The differences in the impacts of biodegradable and nondegradable MPs on heavy metal toxicity to crops are scarcely documented.

Therefore, pot experiments were designed to test the hypothesis that significant differences exist in the impacts of heavy metal toxicity on crops between biodegradable and nondegradable MPs and that these differences depend on the levels of MPs present. PP and PLA were selected as the classic nondegradable and biodegradable MPs, respectively, due to their widespread use in soil–plant systems [1]. Cd was chosen as the representative heavy metal due to its high toxicity and widespread occurrence. To test the hypothesis, this study aimed to compare the impacts of different levels of PP and PLA on Cd uptake by pak choi cultivated in soils with varying Cd concentrations and to elucidate the underlying mechanisms.

## 2. Materials and Methods

### 2.1. Experimental Preparation

Topsoil from a depth of 0–20 cm was sampled in Qingdao, northern China, with two main crops of vegetables and corn. The sampling location information was illustrated in the Supplementary File (Figures S1–S3). Subsamples were sieved through 0.417 mm and 2 cm meshes for basic characteristics analysis and pot experiments. The total Cd contents in the sampled soils were 0.49, 2.52, and 10.1 mg kg^−1^ in the sampled soils, designated as C1, C2, and C3, with corresponding pH values of 7.23, 7.22, and 7.18. The cation exchange capacity (CEC) and dissolved organic carbon (DOC) contents were 20.7, 21.0, and 22.3 c mol kg^−1^ and 1.69, 1.57, and 1.45 g kg^−1^ for C1, C2, and C3, respectively. These soil properties were determined according to the methods described in references [8] and [18].

PP and PLA (8.68–500 μM) were supplied by Sigma Aldrich. Scanning electron microscope (SEM) images and X-ray diffraction (XRD) characterization of these materials are provided in the Supplementary Data. The two types of powdered MP materials were sieved through a 0.5 mm mesh and pretreated using 0.1 mol L^−1^ HNO_3_ and deionized water for the pot experiments. Pak choi (*Brassica campestris* L.) was selected due to its high Cd absorption capacity and its worldwide cultivation under plastic films [17,18].

### 2.2. Pot Experiments

Pot experiments were set up with seven treatments: a control without MPs (CK); 0.1% MPs (PP1/PLA1, mass/mass); 1% MPs (PP2/PLA2, mass/mass); and 5% MPs (PP3/PLA3, mass/mass) in three levels of Cd-polluted soils. A total of 21 groups, each with triplications, were tested. Different amounts of MPs were weighed and thoroughly mixed with the soils. The mixed soils were then transferred into each pot, and water was added until the maximum water holding capacity (MWHC) was reached. The soils were then kept in darkness for two months at room temperature to equilibrate. In total, there were 63 ceramic pots. Each pot contained 200 g of the soil–MP mixture and was planted with 10 surface-sterilized pak choi seeds. Fourteen days after germination, the seedlings were thinned to one per pot. The cultivation process and harvest details were introduced in [8,19].

### 2.3. Sampling and Analysis

After 45 days of growth, the plants were harvested and separated into shoots and roots. Rhizosphere soils were sampled at the time of harvest. The methods for analyzing plant biomass, plant Cd uptake, soil Cd availability, and soil parameters have been detailed in references [8] and [18]. Lipid peroxidation was assessed by measuring the MDA content based on a thiobarbituric acid reaction [20]. The activities of antioxidant enzymes, including SOD, POD, and CAT, were determined using the nitroblue tetrazolium test method, the guaiacol substrate method, and H_2_O_2_, respectively [21].

### 2.4. Statistical Analysis

Statistically significant differences were analyzed using DPP v.20.0 at a significant level of 0.5. regression pathway analysis (RPA) was employed to identify the relationships between shoot Cd uptake and soil parameters.

## 3. Results

### 3.1. Plant Biomass

**Figure 1:**
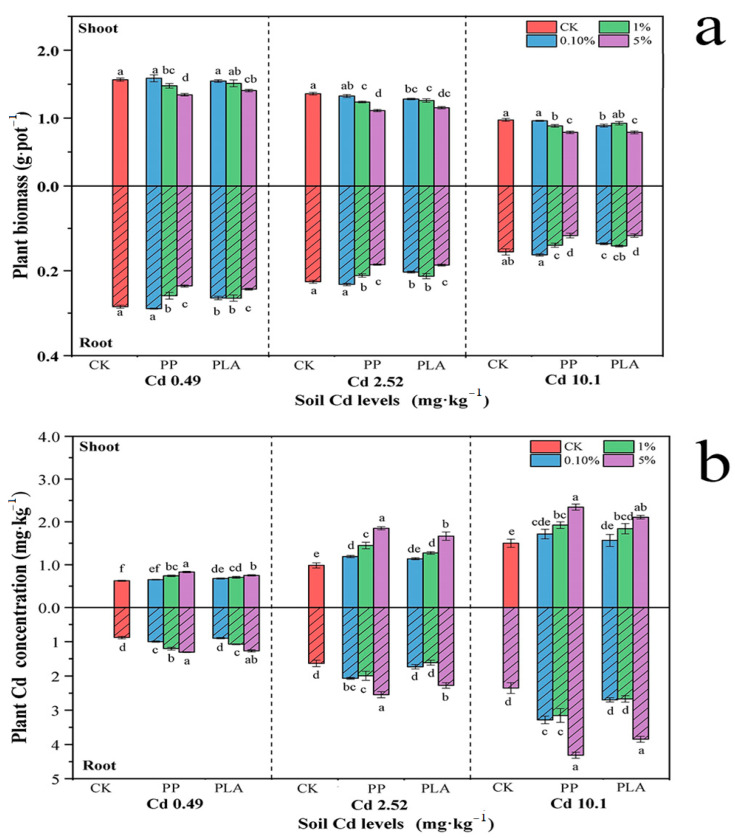
Shoot and root biomass (**a**) dry weights, means ± SD) and Cd uptake (**b**) in plants exposed to coexistence of MPs and Cd in soil. Note: The vertical bars above and within the columns represent standard errors (*n* = 7 × 3). Different lowercase (uppercase) letters indicate significance in the shoot and root biomass of plants and Cd uptake in plants across the amendment treatments at *p* < 0.05 level according to Duncan’s multiple range test; CK—control treatment; 0.1%—treatment of 0.1% MPs loads; 1%—treatment of 1% MPs loads; and 5%—treatment of 5% MPs loads.

The MPs significantly impacted both shoot and root dry weights (except in C1 soil), with the degree of impact highly dependent on the doses and types of MPs across all three Cd levels (Table 1). In the C1 soil, PP significantly decreased plant biomass only at the 5% dose, whereas PLA significantly inhibited plant growth at both the 5% and 1% doses but did not have a significant impact at 0.1% (Figure 1). Notably, PP showed much greater root growth inhibition at the higher doses of 1% and 5% compared to the corresponding doses of PLA, while no significant difference was observed at the lower dose of 0.1%.

### 3.2. Plant Cd Uptake

**Figure 2:**
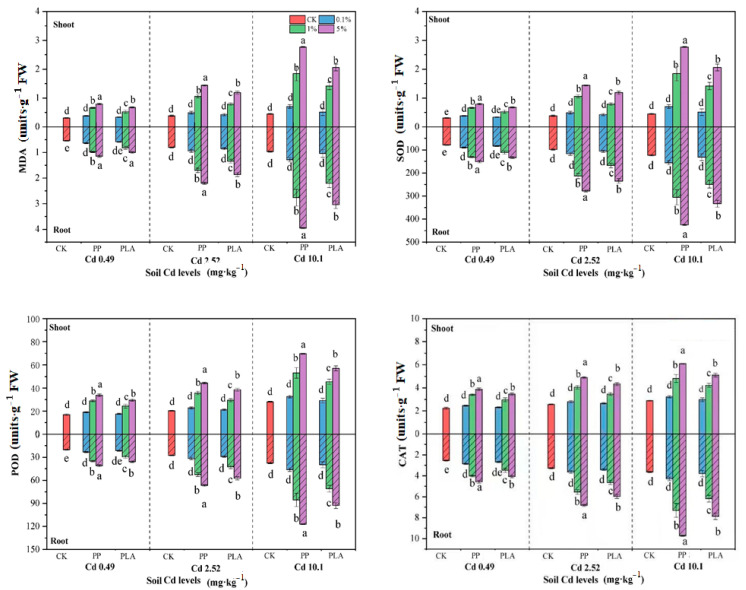
Biomarker contents in plants exposed to coexistence of MPs and Cd in soil. Note: The vertical bars above and within the columns represent standard errors (*n* = 7 × 3). Different lowercase (uppercase) letters indicate significance in the biomarker contents in plants across the amendment treatments at *p* < 0.05 level according to Duncan’s multiple range test; CK—control treatment; 0.1%—treatment of 0.1% MPs loads; 1%—treatment of 1% MPs loads; and 5%—treatment of 5% MPs loads.

Generally, the types and doses of MPs, along with their interactions, significantly altered the plant Cd uptake at different Cd levels, except for the nonsignificant interaction on shoot Cd uptake at the highest Cd level of 10.1 mg kg^−1^ (Table 1). Specifically, compared to CK, PP significantly increased shoot and root Cd accumulations by 20.9–87.9% and 26.9–83.3%, respectively, as the doses increased from 1% to 5% across all Cd levels. Neither PP nor PLA significantly promoted plant Cd accumulation at the 0.1% dose. Notably, PP induced greater increases in the root Cd uptake than PLA in the root Cd uptake at all three doses across all Cd levels, although this increase was not significant at the 5% dose under the 0.49 mg kg^−1^ soil Cd level. In terms of the shoot Cd uptake, this difference was significant only at the 5% dose under 0.49 and 2.52 mg kg^−1^ soil Cd levels (Figure 2).

### 3.3. Biomarker Contents in Plants

MPs significantly impacted the biomarker contents in plants across all Cd-polluted soils, with the impact degree being highly sensitive to both the type and dose of MPs (Table 1). The biomarker contents in both the shoots and roots increased with the MPs dose across all Cd levels, although the increase was not significant at the 0.1% dose relative to the CK. The highest values were observed in the 5% PLA treatment in the C3 soil, with the highest Cd level of 10.1 mg kg^−1^. Specially, PP induced greater increases in biomarker contents than PLA across all levels of MPs and Cd, though this increase was not significant at the 0.1% dose. For instance, the highest MDA contents were 1.44 and 2.20 mmol mg^−1^ of protein for the shoots and roots in the 5% PLA treatment under 10.1 mg kg^−1^ Cd level, which were 1.22 and 1.18 times higher than those in the corresponding 5% PP treatments, respectively.

### 3.4. Bioavailable Cd in Soil

**Figure 3:**
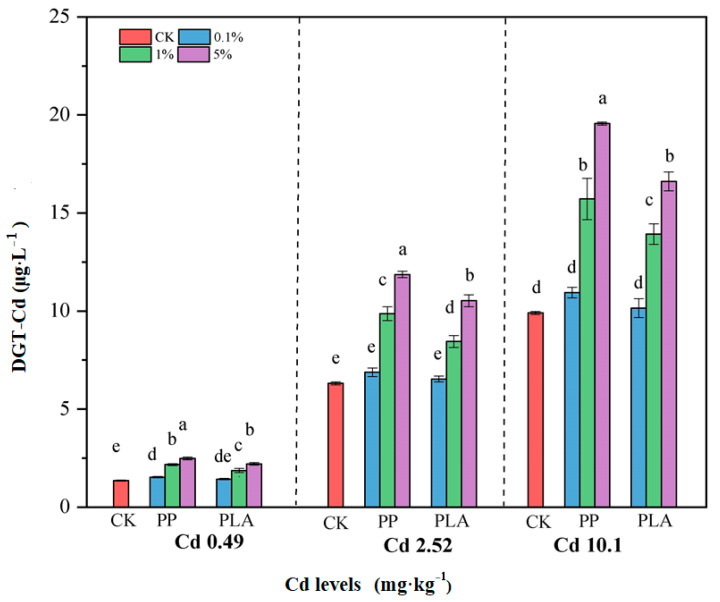
Bioavailable Cd in soil under coexistence of MPs and Cd. Note: The vertical bars above and within the columns represent standard errors (*n* = 7 × 3). Different lowercase (uppercase) letters indicate significance in the bioavailable Cd in soil across the amendment treatments at *p* < 0.05 level according to Duncan’s multiple range test; CK—control treatment; 0.1%—treatment of 0.1% MPs loads; 1%—treatment of 1% MPs loads; and 5%—treatment of 5% MPs loads.

The bioavailable Cd levels in soil were significantly increased by PP (8.98–97.5%), followed by PLA (2.49–67.7%). These increments significantly depended on the types and doses of MPs, as well as their interactions across all three Cd levels (Table 1, Figure 3). The increases in DGT-Cd concentrations induced by both MPs were significantly positively correlated with their doses across all three Cd levels relative to CK, although no significant effect was observed for PLA at the 0.1% dose.

### 3.5. Soil Physicochemical and Microbial Properties

**Figure 4:**
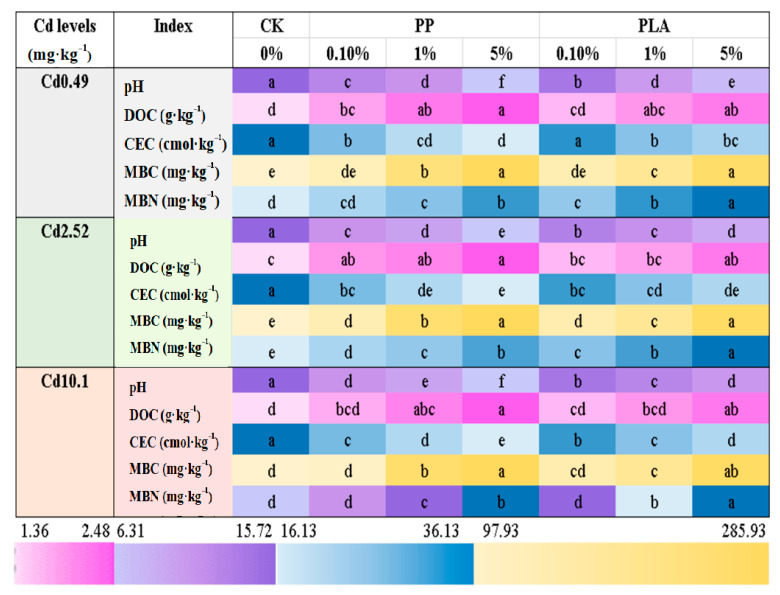
Soil physicochemical and microbial properties with MPs coexisting with Cd. Note: Different lowercase letters indicate significance in the soil physicochemical and microbial properties across the MPs treatments at a *p* < 0.05 level according to Duncan’s multiple range test.

The soil physicochemical properties were significantly affected by both the dose and type of MPs, with some significant response differences to their interactions across all three Cd-polluted soils (Table 1, Figure 4). The type and dose of the MPs did not significantly affect soil DOC across any of the three Cd levels. Both MPs significantly reduced the pH and CEC while significantly increasing the DOC, MBC, and MBN relative to the CK, irrespective of the Cd levels in soil. PP induced greater reductions in the soil pH and CEC and greater increases in the soil DOC compared to PLA. Interestingly, relative to the CK, PP increased the MBC levels by 2.40–89.0%, while PLA increased the MBN levels by 13.1–99.9%.

### 3.6. Relationships between Shoot Cd Uptake and Soil Properties 

**Figure 5:**
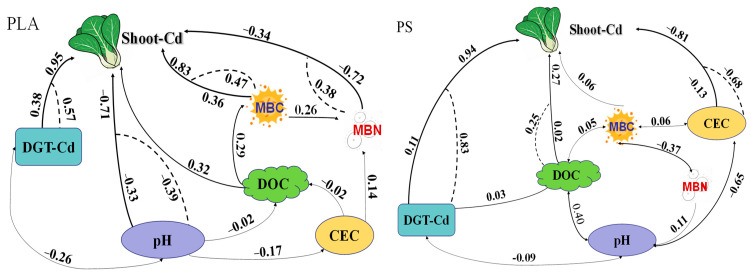
Correlations among soil properties and shoot Cd concentrations based on RPA. Arrows, solid, and dashed lines illustrate the integrated, direct, and indirect impact on the shoot Cd uptakes, respectively.

The RPA results illustrate that pH, CEC, and bioavailable Cd (DGT-Cd) were the main controlling factors for the shoot Cd uptake in both the PP and PLA treatments (Figure 5). Bioavailable Cd positively contributed to shoot Cd uptake, which negatively correlated with pH and CEC. Other soil parameters, including DOC, MBC, and MBN, did not significantly impact the shoot Cd uptake in either the PP or PLA treatments. However, the soil DOC had significant correlations with the soil pH, CEC, MBC and MBN, which also interacted highly with each other. Notably, a significantly negative relationship between the pH and MBC was observed only in the PLA treatments. Therefore, soil parameters such as the DOC, MBC, and MBN may indirectly impact shoot Cd uptake by influencing other soil parameters like pH, CEC, and bioavailable Cd. The indirect influences were −0.173/−0.385, −0.677/−0.590, and 0.830/0.568 for PP/PLA. The relationships suggest that different levels of MPs impact shoot Cd uptake by altering soil variables, which act with direct and/or indirect in the process. The soil pH, CEC, and bioavailable Cd played dominant roles. The microbial properties contributed more to the shoot Cd uptake in the PLA treatments than in the PP treatments.

## 4. Discussion

The results of the present study strongly confirmed the hypothesis, thus demonstrating significant differences in the impacts of heavy metal toxicity to crops between biodegradable and nondegradable MPs. These differences varied with the doses of MPs. Both types of MPs altered plant biomass and plant Cd uptake by impacting the physical–chemical and microbial properties of rhizosphere soil environments across all three Cd levels. The RPA results further explain that the changes in plant Cd uptake were attributed to the direct and/or indirect contributions of soil physicochemical (i.e., pH, CEC, DOC, and Cd bioavailability) and microbial properties (i.e., MBC and MBN).

### 4.1. Phytotoxicity of MPs in Soil–Plant Systems

Previous research has pointed out that MPs can alter toxicity to organisms in soil–plant systems [22]. MPs can act as carriers, thus transporting sorbed heavy metals into organisms and exhibiting different toxic effects. Additionally, MPs can interact with heavy metals, thus altering their uptake in organisms [23] and resulting in different reactivities. While previous studies have investigated the coexistence of MPs and heavy metals, as well as their interactions affecting the toxicity on earthworms in soil–plant systems, few studies are available on the combined toxicity to plants. Several studies have reported the interaction impacts of MPs and heavy metals on soil properties and plant performance [24,25]. For instance, PP did not exert significant phytotoxicity, while PLA inhibited maize plant growth and chlorophyll contents [9]. However, the exact mechanisms of phytotoxicity in plants remain unclear.

This study extends beyond assessing plant growth to analyzing biomarker contents, including MDA, SOD, POD, and CAT. The results have revealed increasing trends in both shoot and root tissues with higher doses of MPs. Supporting earlier research, this finding indicates that biodegradable MPs exhibit less prohibition of plant growth compared to nondegradable MPs [26]. The MDA content, commonly used to detect plant damage from contaminants, is released during lipid peroxidation stimulated by reactive oxygen species (ROS). Higher ROS levels, produced when organisms are injured or stressed, adversely affect plants [27]. Elevated MDA levels suggest that MPs and metal contaminants have harmed the plant bodies and induced toxic effects. Enzymatic activities serve as effective indicators of phytotoxicity [28]. Antioxidant enzymes, crucial for protecting plant cells and maintaining normal cellular activity, help mitigate oxidative stresses and cellular damage [29]. These enzymes, including SOD, CAT, and POD, play key roles in the enzymatic defense system, thus removing oxygen free radicals and managing stress conditions [30]. SOD in plant tissues converts O_2_^−^ into H_2_O_2_, which is then transformed into H_2_O and O_2_ other enzymes such as POD and CAT [31]. Increased enzyme activities are direct responses to heightened levels of superoxide anion radicals [32]. Under stress, plants enhance their ROS levels, thus leading to increased antioxidant enzymes in tissues to counteract oxidative damage. The presence of MPs might reduce plant tolerance to abiotic stress in Cd-polluted soils by impacting the defensive capacities [33]. It is recommended to replace conventional nonbiodegradable plastics with biodegradable alternatives like PLA to control environmental MP pollution, especially in agricultural systems.

### 4.2. Impacts of MPs on Plant Cd Uptake and Soil Cd Bioavailability

The retention and activation capabilities of MPs for soil metals are influenced by their type, shape and size, which can significantly affect heavy metal behavior and accumulation, particularly in soil–plant systems [34]. These effects are primarily due to the varying degradation characteristics of MPs, thus leading to differences in plant cadmium (Cd) uptake and soil Cd availability. The presence of MPs positively impacts both plant Cd uptake and soil Cd bioavailability, thus confirming that MPs enhance soil Cd bioavailability in these systems [8,24,35]. The increased plant Cd uptake, compared to the control (CK), is attributed to the MPs-driven rise in bioavailable Cd content in the soil and the reduction in plant biomass (Figure 1). MPs loaded with HMs easily reintroduce them into soil’s liquid phases, particularly under fluctuating soil conditions. The predominant pathway for plant Cd uptake involves the adsorption of Cd onto root surfaces when MPs and Cd are coexisting in soil–plant systems. MPs enhance Cd bioavailability by reducing the adsorption capabilities and increasing the desorption capacities of Cd from soil to plants [18]. This occurs because the adsorption capacity of Cd onto MPs is lower compared to that of pure soil, which has a higher capacity for Cd adsorption [7]. The effects of coexisting PP MPs on Cd bioavailability in plant–soil systems were highlighted by a previous study, thus indicating that MPs can also enhance Cd exchangeability, lability, bioavailability, and reduce Cd retention in soil through a “dilution effect” and an “activation effect” [35]. Moreover, MPs promote the maintenance of labile Cd mobilization and prevent its stabilization by altering soil conditions and competing for adsorption sites. The distribution of Cd in soil is highly dependent on the soil conditions, thus indicating that changes in soil pH, DOC, and CEC will modify Cd bioavailability. It is implied that MPs influenced Cd uptake by altering both the Cd bioavailability in soil (DGT-Cd) and the soil parameters, which interact with each other (Figure 5). In the future, it will be valuable to explore the distribution and translocation of Cd at both the tissue and cellular levels within plants, as well as its transfer across the interfaces of the soil–root–shoot system.

### 4.3. Alterations by MPs in Properties of Cd-Contaminated Soils

Numerous studies have reported that various nondegradable and biodegradable types of MPs accumulate heavy metals such as Cr, Ni, Cu, Cd, Pb, and Hg, thereby altering soil properties [36,37]. In the present study, PP differed significantly from PLA in both the soil physicochemical and microbial properties, although both types of MPs had significant impacts. This distinction is supported by previous research indicating that the impacts of nonbiodegradable MPs on soil properties are generally modest, whereas those of biodegradable MPs are more significant [38]. For instance, nonbiodegradable MPs often result in lower pH levels compared to biodegradable MPs [9,35], which is a trend also observed in the present study, where PP caused greater reductions than PLA. Soil pH is a crucial factor controlling the bioavailability and mobility of soil Cd [39]. In soils with lower pH values, Cd may exhibit higher mobility and bioavailability, thus potentially enhancing Cd accumulation in plants [17]. This difference can be attributed to the distinct biodegradation characteristics of the two MPs. Biodegradable PLA plastic, serving as an alternative to nondegradable MPs, has been shown to degrade at an average degradation rate of 7675 Mw per week in a Costa Rican soil under field conditions [40]. Due to their smaller sizes, PLA likely degrade faster than PLA in the same condition, thus exerting a greater impact on soil properties than nondegradable MPs [8,26]. Moreover, the shapes, polymer types, and surface characteristics of biodegradable PLA may significantly after natural biodegradation [41], thus resulting in more complex interactions affecting soil Cd. The RDA results underscore the interconnectedness among these soil parameters, thus suggesting that MPs (microplastics) have multifaceted effects on soil characteristics beyond pH alone and potentially influencing plant Cd uptake (Figure 5). Previous studies have also indicated that the mineralization of PLA may lower soil pH by releasing lactic acid [9,35]. Soil, being a complex medium influenced by various environmental factors such as organic and inorganic ligands, CEC, and microbial conditions, can significantly impact the transfer of Cd from soil to plants. In this study, PP and PLA exerted distinct influences on soil properties and the behavior of heavy metals in soil–plant systems. Additionally, MPs can alter soil DOC contents by affecting soil aggregation, thereby stimulating soil microbes to secrete substances [42]. The trend of increasing DOC contents, along with positive relationships between DOC contents and/or MBC/MBN, implies a beneficial impact on soil microbial properties due to the presence of MPs. However, slight and insignificant decreases in the DOC were observed with low doses of nondegradable MPs (<5%) [43]. Furthermore, rising pH tends to increase DOC contents in soil, as supported by the relationships among pH, CEC, and DOC (Figure 5). Soil DOC plays a significant role in supplying carbon to soil microbes, thereby potentially enhancing soil microbial functions [44]. Therefore, alterations induced by MPs in the soil physicochemical parameters observed in this study promote microbial activity and improve the soil microenvironment [8,9,45]. Interestingly, divergent correlations between pH and MBC were observed with the two types of MPs in this study, thus suggesting a more dynamic and fluctuating soil environment in PLA-treated soils compared to those in PP-treated soils. This finding strongly supports the previously mentioned potential mechanisms underlying the varying impacts of PLA and PP on plant and soil parameters.

Overall, nondegradable PP exhibited greater phytotoxicity and had more negative effects on plant growth while also contributing more significantly to plant Cd uptake compared to PLA. Consequently, biodegradable PLA plastics are recommended as an alternative to nonbiodegradable plastics, especially in agricultural systems facing higher Cd contamination. However, attention must be given to the by-products generated during PLA degradation (such as additives, plasticizers, and minerals), which have been reported to negatively impact soil properties and plant growth. Therefore, it is essential to thoroughly investigate the long-term effects of biodegradable MPs in soil–plant systems, particularly under field conditions. Further research is needed to better understand the molecular mechanisms affecting plant cells and soil functions.

## 5. Conclusions

This study examined the interactions between MPs and Cd on plant biomarker (MDA and antioxidant enzymes). MPs significantly influenced plant biomass and biomarker levels across all Cd levels, with the extent of impact being highly sensitive to both the type and dosage of MPs. Compared to nondegradable PP, biodegradable PLA generally had a lesser influence on plant phytotoxicity, Cd availability, and soil properties. The RPA results indicate that MPs increased shoot Cd uptake by modifying soil physical–chemical and microbial characteristics. While pH, CEC, and Cd bioavailability were identified as crucial soil variables, no single factor alone explained the plant Cd uptake mechanisms comprehensively. Biodegradable PLA plastics are recommended as superior alternatives to nondegradable plastics for environmental protection, particularly in agricultural systems facing higher Cd contamination. However, the long-term negative impacts of the by-products produced during PLA degradation require attention in soil–plant systems, especially under field conditions.

## Figures and Tables

**Table 1:** Significance levels of MPs types, doses, and their interactions on measured variables in three samples according to two-way ANOVA analysis.

Variables	*F* Values and Significant Levels								
	Type			Dose			Type × Dose		
	Cd1	Cd2	Cd3	Cd1	Cd2	Cd3	Cd1	Cd2	Cd3
Shoot biomass	1.29 ^ns^	0.44 ^ns^	1.12 ^ns^	47.3 ***	169 ***	96.9 ***	2.92 ^ns^	5.89 **	8.56 **
Root biomass	2.50 ^ns^	21.1 ***	8.88 **	109 ***	144 ***	68.4 ***	15.6 ***	27.2 ***	11.0 ***
Shoot MDA	48.7 ***	69.2 ***	47.5 ***	361 ***	599 ***	367 ***	8.84 ***	14.5 ***	10.1 ***
Root MDA	49.4 ***	69.2 ***	47.6 ***	367 ***	601 ***	368 ***	8.94 **	14.5 ***	10.1 ***
Shoot SOD	49.0 ***	69.0 ***	47.6 ***	364 ***	599 ***	367 ***	8.88 **	14.5 ***	10.1 ***
Root SOD	49.0 ***	69.0 ***	47.6 ***	364 ***	599 ***	367 ***	8.88 **	14.5 ***	10.1 ***
Shoot POD	42.9 ***	69.0 ***	47.6 ***	308 ***	599 ***	367 ***	7.78 **	14.5 ***	10.1 ***
Root POD	49.0 ***	69.0 ***	47.6 ***	364 ***	599 ***	367 ***	8.88 **	14.5 ***	10.1 ***
Shoot CAT	43.0 ***	69.0 ***	47.6 ***	309 ***	599 ***	368 ***	7.80 **	14.5 ***	10.1 ***
Root CAT	48.9 ***	68.9 ***	47.6 ***	364 ***	597 ***	367 ***	8.86 **	14.4 ***	10.1 ***
Shoot Cd uptake	12.6 **	19.0 ***	8.33 *	131 ***	200 ***	61.8 ***	13.3 ***	3.69 *	1.50 ^ns^
Root Cd uptake	32.0 ***	48.5 ***	54.0 ***	249 ***	88.5 ***	192 ***	5.92 **	5.79 **	6.24 **
DGT-Cd	49.0 ***	69.0 ***	47.6 ***	364 ***	599 ***	367 ***	8.88 **	14.5 ***	10.1 ***
pH	18.1 ***	360 ***	1003 ***	680 ***	1459 ***	1392 ***	11.6 ***	51.5 ***	122 ***
CEC	38.0 ***	23.2 ***	40.3 ***	107 ***	113 ***	179 ***	4.39 *	2.61 ^ns^	4.62 *
DOC	6.30 *	5.28 *	5.00 *	38.6 ***	24.2 ***	30.9 ***	0.71 ^ns^	0.61 ^ns^	1.08 ^ns^
MBC	10.3 **	27.8 ***	30.4 ***	265 ***	388 ***	362 ***	7.99 **	23.2 ***	36.8 ***
MBN	27.9 ***	53.5 ***	34.3 ***	110 ***	209 ***	274 ***	4.87 *	6.03 **	4.83 *

Note: *, **, ***, and ^ns^ represent significant levels of *p* < 0.05, 0.01, 0.001, and nonsignificant effect.

## Data Availability

Data will be made available on request.

## Supplementary Materials

The following supporting information can be downloaded at: https://www.mdpi.com/article/10.3390/toxics12070473/s1, Figure S1: Scanning electron microscope (SEM) images of MPs; Figure S2: X-ray Diffraction (XRD) characterization of MPs; Figure S3: Sampling location.
