# Supplementary material for: Plant Cadmium Toxicity and Biomarkers Are Differentially Modulated by Degradable and Nondegradable Microplastics in Soil
Source: Toxics. 2024 Jun 29;12(7):473. doi: 10.3390/toxics12070473 (PMC11280994; doi:10.3390/toxics12070473)
Supplement: Supplementary file 1 [file toxics-12-00473-s001.zip › toxics-3066978-supplementary.pdf]

SUPPLEMENTARY MATERIALS

Characterization of the microplastics used in this study

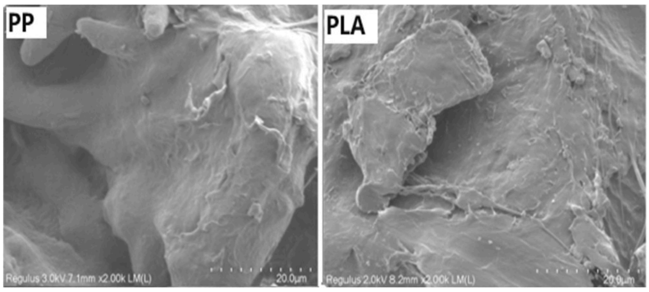

Figure 1. Scanning electron microscope (SEM) images of MPs

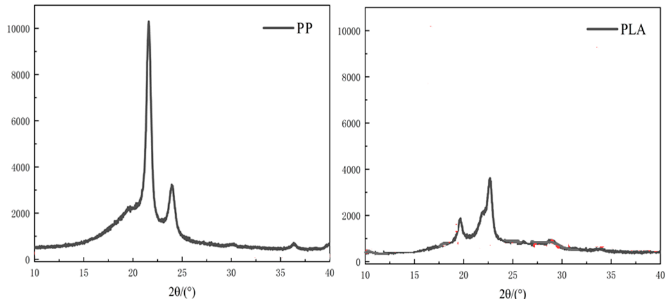

Figure 2. X-ray Diffraction (XRD) characterization of MPs

Sampling location map

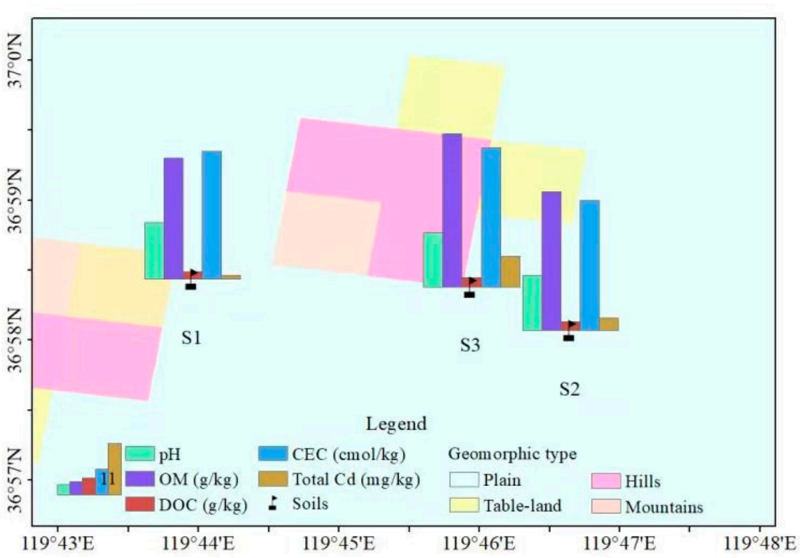

Figure 3. Sampling location
